# Getting to the Bottom of Face Processing. Species-Specific Inversion Effects for Faces and Behinds in Humans and Chimpanzees (*Pan Troglodytes*)

**DOI:** 10.1371/journal.pone.0165357

**Published:** 2016-11-30

**Authors:** Mariska E. Kret, Masaki Tomonaga

**Affiliations:** 1 Leiden University, Institute of Psychology, the Cognitive Psychology Unit, Leiden, the Netherlands; 2 Leiden Institute for Brain and Cognition (LIBC), Leiden, the Netherlands; 3 Primate Research Institute, Kyoto University, Inuyama, Aichi, Japan; Harvard Medical School, UNITED STATES

## Abstract

For social species such as primates, the recognition of conspecifics is crucial for their survival. As demonstrated by the ‘face inversion effect’, humans are experts in recognizing faces and unlike objects, recognize their identity by processing it configurally. The human face, with its distinct features such as eye-whites, eyebrows, red lips and cheeks signals emotions, intentions, health and sexual attraction and, as we will show here, shares important features with the primate behind. Chimpanzee females show a swelling and reddening of the anogenital region around the time of ovulation. This provides an important socio-sexual signal for group members, who can identify individuals by their behinds. We hypothesized that chimpanzees process behinds configurally in a way humans process faces. In four different delayed matching-to-sample tasks with upright and inverted body parts, we show that humans demonstrate a face, but not a behind inversion effect and that chimpanzees show a behind, but no clear face inversion effect. The findings suggest an evolutionary shift in socio-sexual signalling function from behinds to faces, two hairless, symmetrical and attractive body parts, which might have attuned the human brain to process faces, and the human face to become more behind-like.

## Introduction

For group-living animals, primates included, the recognition of conspecifics is crucial for their survival. Humans have specialized brain areas to recognize faces[1] and whole bodies[2–5] and their expertise in face recognition is demonstrated by the ‘inversion effect’, showing that faces and whole bodies, but not objects, are recognized configurally rather than by their parts[6–8]. Importantly, their recognition is *disproportionally* impaired, relative to objects such as houses or cars, when they are seen inverted rather than upright[6]. Conclusive evidence has shown that this effect is primarily due to a disruption in the processing of configural, rather than featural, information in faces [e.g., [9–13]. The face inversion effect has been observed in chimpanzees too, and although not all chimpanzees show this effect at all times[14, 15], overall there is evidence that configural processing is a critical element of efficient face detection in chimpanzees as well[16, 17]. Thus, effects of inversion have been observed for faces and whole bodies, but are generally not found for individual body parts[18]. Intriguingly, previous studies included almost all body parts, except the most obvious one, which is the behind, as we will outline below.

Previous research has shown that in recognizing each other, chimpanzees do not rely on the face alone[14], but also easily recognize each other by their behinds[19]. Most non-human female primates, chimpanzees included, show a swelling and reddening of the anogenital region around the time of ovulation[20]. At some point during human evolution, these changes in size and color along the menstrual cycle have disappeared, and large quantities of ‘permanent’ adipose tissue on the behind emerged[21, 22]. Possibly, this became more adaptive when our species started to walk upright, or to hide oestrus as to be attractive for males throughout the menstrual cycle and foster pair bond formation and shared caring for offspring. To date, it is not known how behinds as compared to faces are recognized in humans and their closest relatives, but this knowledge can enhance our understanding of the evolution of face processing, as we will argue below.

Face recognition plays an incredibly important role in the survival of animals living in social groups, including humans and chimpanzees. The changeable properties of faces like expression and gaze, display emotions and intentions and are used by observers to predict behavior[23]. The more or less invariant properties of faces are used for identification and display physical characteristics, including sex, age and attractiveness[1].

The primate behind is unlike any other body part. In chimpanzee females, as in other catarrhine primate species, the anogenital region swells, becomes shiny and smooth and reddens around the time of their ovulation. Male mating interest is positively correlated with these changes, but the changes do not go unnoticed by competing females either[20]. Besides accumulating adipose tissue on their behinds, which might have been a sign of fitness in harsh savanna conditions, human females also developed relatively large breasts and are unique amongst primates to develop these already before their first pregnancy[22]. It has been suggested that breasts evolved to resemble the bottom, being more visible when walking upright[21, 24]. Also, humans, especially females, developed reddened and thicker lips and fattier faces as compared to chimpanzees, which is also a sign of beauty and these features are often accentuated with make-up[25]. (On a side note, from personal observation it seems that bonobo’s fall in between chimpanzees and humans in terms of lip color). Thus, human faces share important features with the ancient primate behind. And there are even more shared properties. Faces and behinds have a reliable structure across individuals and are ubiquitous in the environment, ensuring high levels of exposure. Furthermore, faces and behinds are both symmetrical, an important characteristic both in lower and higher level visual processing[26, 27]. Also, the correct interpretation of the conveyed information by faces and behinds, including identity, fitness and fertility, is crucial for reproductive success[20, 28, 29].

Research has suggested that colour vision in primates was selected for discriminating the spectral modulations on the skin of conspecifics, presumably for the purpose of recognizing emotional states and socio-sexual signals including threat displays and swellings[30]. Whereas humans are predominantly furless, the face and behind are some of the few hairless areas on the body of a chimpanzee. The expansion of areas on the body showing bare skin over human evolution is one potential way of utilizing this medium for signalling. Therefore, the visibility of the skin and its colouration might contribute to the signalling function of faces and behinds.

**Fig 1:**
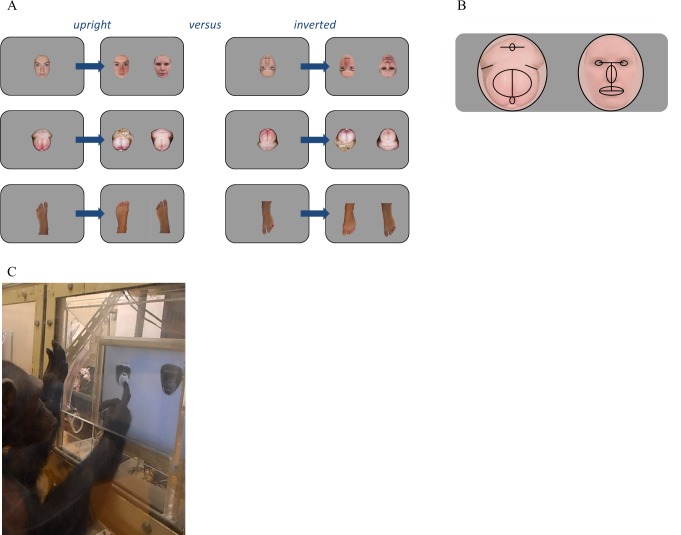
**A.** Stimulus examples shown in Experiment 1 and 3. What is shown are upright examples on the left panel and inverted examples on the right. The different stimulus categories are shown in each row, with human faces in the first row, chimpanzee behinds in the second, and human feet in the third. The correct response in each row, starting at the top, is left, right, and right (the same response is correct for both the upright and inverted examples). **B.** Chimpanzee behinds (depicted on the left), contain specific features like human faces (depicted on the right). The black lines highlight those features. **C.** An example of a chimpanzee participant conducting the task with desaturated images. Experiments 2 and 4 used desaturated images.

Whether humans and chimpanzees process behinds the way they process faces is thus far unknown. If so, they should not only be able to recognize individuals by these body parts, but also process them configurally, and show a *behind inversion effect*. This would show which body parts or information sources different species rely on when recognizing individuals, including potential mates or competitors. In the current study, we therefore investigated whether behinds, like faces, are processed configurally, and whether the putative behind inversion effect is enhanced in chimpanzees, where, in contrast to humans, the female genital region changes in size and colour over the menstrual cycle. Given that human and chimpanzee faces and behinds are furless, we predicted that the presence of colour (red) would foster the recognition process. These questions were addressed in four different experiments, two in humans and two in chimpanzees, where both species matched images of faces, behinds and, as a control condition feet (Body Part). The images showed humans or chimpanzees (Stimulus Species) in upright or inverted position (Body Parts Orientation; see Fig 1).

In Experiment 1, human participants were requested to match faces, behinds and feet of both humans and chimpanzees. We tested our hypothesis that they would show a face inversion effect[6–8] and explored the possibility of a behind inversion effect. On the one hand, humans show a body inversion effect[2, 8], and thus, a behind inversion effect seems plausible. But on the other hand, there is not much literature on how humans process behinds, and the behinds that we see in our daily life are usually covered, making it equally likely that humans would not show a behind inversion effect. Experiment 2 tests the same hypotheses but with the additional factor of color. Orientation has been proven to be dominant over color when it comes to face processing[31]. That said, activity in brain areas involved in face processing is boosted when faces are presented in color[32, 33]. We predict that humans will still show a face inversion effect when faces are presented in greyscale, but that the effect is somewhat blunted as compared to Experiment 1 where faces are shown in color. In Experiment 3 we test our hypothesis based on previous observations that chimpanzees will show a face inversion effect[14–17]. Given the high socio-sexual relevance of behinds, we predict that they will show a behind inversion effect too. In Experiment 4 we investigate whether this putative behind inversion effect disappears when turning the pictures into greyscale, which presumably to a large extent will remove the sexual signal[30, 34].

## Method

### Method Experiment 1

#### Participants Experiment 1

In Experiment 1, 49 female (18–28 years, *M* = 21, SD = 3) and 58 male students (19–41 years, *M* = 24, SD = 5) of the University of Amsterdam participated. They were recruited through the university’s online participant portal. Additional male participants were approached in the hallway. In the short online announcement, potential participants read that the study looked at how humans processed faces and other body parts including behinds. Participants had to be at least 18 years old in order to be allowed to participate.

The sample size is sufficiently large as compared to previous studies[6–13, 18], and the exact number of participants resulted from the amount that showed up during the two weeks where we had the lab reserved. Four participants had to be excluded because they performed at chance level, also in the upright face condition, and had extremely fast reaction times. This left the total of participants included at N = 103.

The experimental procedures were in accordance with the Declaration of Helsinki and approved by the Ethical Committee of the Faculty of Behavioral and Social Sciences of the University of Amsterdam (EC No. 2013-WOP-2700). Participants provided written informed consent prior to the experiment and received full debriefing and performance-contingent pay-out on completing the study.

#### Stimulus Materials Experiment 1

The stimuli photographs were taken under well-lit conditions without flash. The stimulus material included pictures from behinds, faces, feet of three human and three chimpanzee females. The individuals in the chimpanzee photographs lived at the Kumamoto Primate Sanctuary, Japan, and were obtained from Mori et al.[34]. The pictures were taken at maximal tumescence. The photos of human behinds depicted the anogenital regions and were taken from the same angle as the chimpanzee pictures, to resemble them as closely as possible. Before the pictures were taken, we asked the women (aged 28–29 years old) to remove their pubic hair as the presence of hair would make the discrimination trivial without looking at the configural information in the anogenital region. They signed an informed consent and allowed us to use these photographs anonymously for the purpose of this study.

To isolate and invert the body parts, the pictures were edited in Photoshop and resized to 2264 x 1584 pixels. Also, the luminance of each image was set to the average. Three different photographs of three different individuals were used. None of the individuals shown in the stimuli were familiar to any of the participants and participants were notified that the presented behinds were not from the same individuals as from the faces. Stimuli included N = 108 unique pictures: (Body Part (face, behind, foot) x Species (human, chimpanzee) x Body Part Orientation (upright, inverted) x three individuals x three exemplars) = 3 x 2 x 2 x 3 x 3 = 108. Stimuli were presented in full colour.

#### Procedure Experiment 1

The task used was a delayed matching-to-sample task and took about ten minutes to complete. A trial began with the presentation of a fixation cross and subsequently, a photograph, i.e., the to-be remembered 'sample', which was presented for two seconds in the middle of the computer screen (sized), slightly above the centre. Then this photograph disappeared from the screen and immediately two new photographs were presented on the left and right side of the screen that stayed on the screen until a response had been given. One of these depicted an individual with the same identity and body part as the photograph that was displayed before, i.e., the ‘match’, and the other photo depicted the same category, but from a different individual. Participants sat in a dimly-lit testing booth at a distance of 60cm from the computer screen and had to identify the matching photograph by pressing the button on the button box that corresponded with their choice. They were requested to do so as fast as possible, and not to think too long before responding. For instance: participants would see a photo of the face of chimpanzee A. After this, two new photos would be displayed: a different photo of the face of chimpanzee A, next to a photo of the face of chimpanzee B or C. In this case the correct response would be the photo of chimpanzee A. We on purpose decided that the match should not be 100% identical to the sample as has sometimes been done in previous studies, but should show a picture of the same individual but taken at a different moment. This way, we prevented that participants matched on the level of low-level features of the image rather than on the higher level of identity. See Fig 1 for examples. Instructions were further kept to a minimum, trying to keep the procedure as similar to the chimpanzee procedure as possible. The task for chimpanzees took about ten minutes and therefore human participants completed 170 random trials which corresponded to approximately 10 minutes. After this task, they took part in an unrelated experiment which took about 20 minutes[35]. They were rewarded 5 euro for their participation in these two studies.

#### Statistical Analyses Experiment 1

Reaction times of the correct trials were included in the analysis, if they fell within the range of +/-2 SDs from the individual mean[35–37]. Data was analyzed in a generalized mixed multi-level model implemented in IBM Statistics 20 with trials nested in individuals and a random intercept for individual[37]. Thus, the multilevel structure was defined by the different trials, nested within participants. Fixed predictors included ‘Body Part’, ‘Body Part Orientation’ and ‘Species Stimulus’. Picture category (Body Part (Face = -1, Foot = 0 and Behind = 1); Body Part Orientation (Upright = 1, Inverted = -1); Stimulus Species (Chimpanzee = -1 and Human = 1) and interactions between picture categories were included as fixed factors along with random intercepts for each individual. As the reaction time data was skewed, a gamma probability distribution was selected with a Log link function[37].

It is recommended that when the errors and reaction time (RT) point into the same direction, the focus should be on the RT analysis, unless the percentage of errors is high enough, e.g., more than 15%[38]. In the current study, error rates and RTs pointed into the same direction and the error rate was too low to analyse. Specifically, in Experiment 1 and 2, overall accuracy was 83% (SD = 0.08) and 90% of the participants performed 100% correct in at least one of the experimental conditions. For these reasons, we focus on the reaction times. The means and standard deviations for the accuracy data can be found in S1 Table.

For conciseness, we only report effects that include the factor Body Part Orientation, but all results can be found in S2–S5 Tables.

### Method Experiment 2

#### Participants Experiment 2

Experiment 2 included 61 female (18–25 years old, *M* = 21, SD = 2) and 46 male students (18–37 years old, *M* = 23, SD = 6) of the University of Amsterdam participated. One participant had to be excluded due to low recognition rates and extreme response times, making the sample count 106 participants.

#### Stimulus Materials Experiment 2

The stimuli photographs were the same as in Experiment 1and included pictures from behinds, faces, feet of three human and three chimpanzee females.

In contrast to Experiment 1, the stimuli were turned into greyscale and pictures of cars were included as an additional stimulus category. There were N = 126 unique pictures (Body Part (face, behind, foot) x Species (human, chimpanzee) x Body Part Orientation (upright, inverted) x three individuals x three exemplars) = 3 x 2 x 2 x 3 x 3 = 108, and Orientation (upright, inverted) x three cars x three exemplars = 2 x 3 x 3 = 18 pictures of cars).

#### Procedure Experiment 2

The procedure was the same as in Experiment 1, but the total number of trials was increased with the addition of one more stimulus category and consisted of 180 trials.

#### Statistical Analyses Experiment 2

The procedure was the same as in Experiment 1. The category ‘car’ was analysed separately.

### Method Experiment 3

#### Participants Experiments 3

Five chimpanzees from the Kyoto University Primate Research Institute took part in Experiments 3–4. At the time of testing, the four females (12–36 years, *M* = 29, SD = 9.9) and the one male (12 years old, and the only male individual available for testing) lived within a group of fourteen individuals in an enriched environment with a 700m^2^ outdoor compound and an attached indoor residence that was illuminated during day-time. The outdoor compound was equipped with climbing frames, ropes, small streams, and various tree species. Access to the outdoor area was available to them every other day during the day. Meals included a wide variety of fresh fruits and vegetables fed throughout the day supplemented with nutritionally balanced biscuits (twice daily) and water available ad libitum. The chimpanzees have been familiar with humans since birth, interacting with them on a daily basis and have taken part in cognitive experiments including matching to sample tasks since youth (for example, see[15–17, 39]. For the daily experiments, the chimpanzees left the group voluntarily on the request of experimenters, moved into the experimental booth, and moved back to the group after the completion of experiments (approx. 1 hour). The two 12-year olds were tested together with their mothers who were participants as well. The care and use of the chimpanzees adhered to the 3^rd^ edition of the Guide for the Care and Use of Laboratory Primates issued by Primate Research Institute, Kyoto University (KUPRI) in 2010, which is compatible with the guidelines of the National Institute of Health in the United States of America. This study was approved by the Animal Welfare and Animal Care Committee of KUPRI and by the Animal Research Committee of Kyoto University (#2012–041, 2012–147, and #2012–148). All procedures adhered to the Japanese Act on Welfare and Management of Animals.

#### Stimuli Experiment 3

The stimulus material was identical to Experiment 1 and 2.

#### Procedure Experiment 3

The chimpanzee participants started with eight training sessions that were spread over two days. Stimuli consisted of Japanese castles (they can see one from their outdoor compound). When performance was at 80% correct, the first session of Experiment 3 or 4 was started, in counter-balanced order. The experiments were spread over ten sessions composed of 72 trials, thus each individual completed 720 trials in total. Chimpanzees received a piece of apple after a correct response. In case of an incorrect response, the trial was replayed, showing the correct answer. No action from the chimpanzee was required during this re-play trial.

The task for the chimpanzees was similar to that of humans (Experiment 1–2), but the sessions were conducted inside an experimental booth designed for chimpanzees (1.8×2.15×1.75 m). A 21-inch color CRT monitor (NEC PC-KH2021) with a capacitive touchscreen device (Microtouch SM-T2) was installed 15 cm from the floor on one side of the booth. Touching the monitor surface with a finger was defined as a response. The screen was protected from deterioration by a transparent Plexiglas panel fitted with an armhole (10×47 cm) that allowed hand contact with the CRT. The resolution of the monitor was 640×400 pixels. One hundred pixels corresponded to 55 mm. Chimpanzees sat at the screen at approximately a distance of 40 cm.

#### Statistical Analyses Experiment 3

The statistical procedure was the same as in Experiment 1 and 2 with the exception that we used a multilevel model with the different trials, nested within dates, nested within sessions, nested within individuals.

### Method Experiment 4

#### Participants Experiments 4

The participants were the same as in Experiment 3.

#### Stimuli Experiment 4

The stimulus material was identical to Experiment 2.

#### Procedure Experiment 4

For the exact same procedure, see Experiment 3. Chimpanzees completed 84 trials per session and completed ten sessions, thus completing 840 trials in total.

#### Statistical Analyses Experiment 4

The statistical procedure was the same as in Experiment 3.

## Results

### Experiment 1 Human participants, coloured stimuli

**Fig 2:**
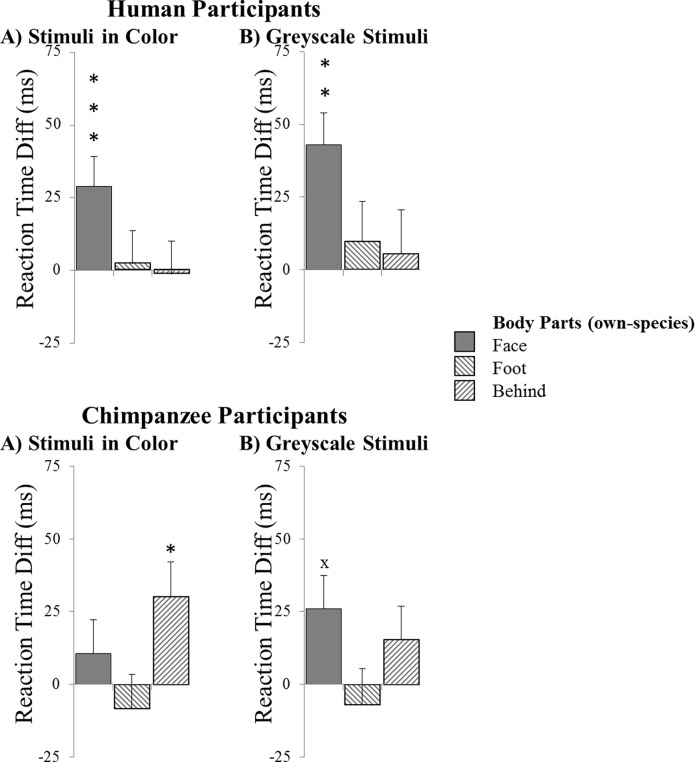
Reaction times for human and chimpanzee participants to conspecific body parts. Reaction time is presented as a difference score (Diff), i.e., Reaction times for Inverted minus Upright stimuli. **A.** Experiment 1, human participants, stimuli in color. **B.** Experiment 2, human participants, desaturated stimuli. **C.** Experiment 3, chimpanzee participants, stimuli in color. **D.** Experiment 4, chimpanzee participants, desaturated stimuli. x *p* < .1; * *p* < .05; ** *p* < .01. Error bars represent the standard error of the mean.

In human participants, an interaction between Body Part (face, behind, foot) * Body Part Orientation (upright, inverted) * Stimulus Species (human, chimpanzee), *F*(2, 6.834) = 4.319, *p* = .013, demonstrated a specific inversion effect for human faces, *t*(6.834) = 2.927, *p* = .003, Fig 2A, but not for human and chimpanzee behinds and feet or chimpanzee faces (*p*s > .173). See S2 Table.

### Experiment 2 Human participants, desaturated stimuli

In a second experiment, we aimed to investigate the effect of desaturating the stimuli. When the stimuli were turned into greyscale, the three- and four-way interactions that we observed in Experiment 1 were rendered insignificant (*p*s > .228). However, a planned comparison showed that, in line with Experiment 1, a face inversion effect was observed for human faces *t* = 2.185, *p* = .029 (Fig 2B). See S3 Table. Thus, these two experiments showed that humans recognize feet and behinds and chimpanzee faces by the parts rather than as a whole. In contrast, human faces, presented either in color or in greyscale are identified configurally.

### Experiment 3 Chimpanzee participants, colored stimuli

A third experiment tested five chimpanzees (one male). The results show a trend towards an interaction between Body Part * Body Part Orientation *F*(2, 2.600) = 2.809, *p* = .06, demonstrating an inversion effect for behinds *t* = 21.161, *p* = .038; Fig 2C and not for faces (*p* = .270) or feet (*p* = .228). See S4 Table.

### Experiment 4 Chimpanzee participants, desaturated stimuli

As was the case in humans, grey-scaling the images rendered the interaction between Body Part and Body Part Orientation insignificant (*p* = .241). See S5 Table. However, we again made two planned comparisons to specifically investigate effects of inversion on the processing of conspecific faces and behinds. In contrast to Experiment 3, an inversion effect on processing behinds was not observed (*p* = .474; Fig 2D), but a trend towards a significant face inversion effect was observed (*t* = 1.664, *p* = .096).

To recapitulate, we replicate the well-known face inversion effect in humans and show that chimpanzees demonstrate an effect of inversion when processing behinds, exclusively when presented in full color.

## Discussion

The current study shows chimpanzee’s expertise in recognizing behinds and suggests they process the bright pink sex swellings of female chimpanzees configurally and in a similar way as humans process faces. The female chimpanzee’s behind has a very high socio-sexual signaling function and the changes in size and color over the menstrual cycle reflect fertility. For that reason, it is important for conspecifics to be able to quickly detect this signal in the environment, but at the same time, it is vital to know who the behind belongs to[19]. For male chimpanzees this is relevant to prevent inbreeding. In turn, for female chimpanzees it is relevant to be aware of competing females to protect their own mating success.

The current study replicates previous research on the face inversion effect in humans, demonstrating that they process faces configurally[2]. In line with our hypothesis, the face inversion effect was dampened when faces were turned into greyscale, but still strongly significant, which is in line with previous research in humans showing that orientation is more important than color when it comes to processing human faces[31]. Also without color, the human face contains many high contrasting features such as eye whites, a prominent nose and lips and eyebrows. Although facial color can provide important social information, such as about emotions and health, there are also minor alterations over the menstrual cycle [40]. However, these small changes are beyond any comparison with the rich coloration of the chimpanzee behind where the alterations are much more obvious. In chimpanzees, the relevance of color for processing behinds is reflected in the *absence* of the behind inversion effect when pictures of behinds were presented in greyscale. In real life, the size and color of the swelling change in synchrony over the menstrual cycle. Thus, a full swelling around estrus is always redder than the female behind half a cycle later. It is therefore possible that due to the un-naturalistic mismatch between color (grey) and size (full swelling), these behinds were processed as objects, i.e., identified by the parts rather than as a whole.

Like humans, great apes are optimally equipped to process color and the spectral sensitivity of the cones in their retinas is ideal for discriminating both density of hemoglobin and oxygen saturation of the blood[30]. Also, the brain areas specialized in processing faces and bodies possess unique neural wiring to effectively process color[32, 33]. Once developed over the course of evolution, color vision (and especially trichromatic color perception) proceeded to impose a selective pressure on certain external traits such as the pink female sexual swelling in chimpanzees and the red lips and cheeks in humans.

A limitation of this study is the low number of individuals in the chimpanzee sample. Although this is common in most primate research and is largely compensated for by the large number of trials per individual, it is possible that effects would have been stronger had we been able to test a larger sample. Moreover, the chimpanzees in our sample were adolescents and adults and we can therefore only speculate about whether this specialization in processing behinds is inborn or related to expertise and emerged sometime during the developmental trajectory. In humans, the specialization for faces occurs already in the first couple of months of life[41]. In fact, already from birth, infants are interested in other people’s faces and eyes and make eye-contact[42]. The making of eye-contact is also facilitated in our species as walking upright freed the hands of parents, allowing them to carry their babies in their arms more often[43]. In contrast, chimpanzees are knuckle-walkers and carry their infants on their belly or back. For them, the swellings become particularly relevant only around puberty. The swellings also appear around that time, i.e., around the age of 10, and at that age, the color of the face changes from pink to a permanent black tint, reducing the contrast with the rest of the body[20]. The swellings stand out enormously in terms of color, size, smoothness and shininess and have a much stronger socio-sexual signaling function in the chimpanzee than the face. Future experiments with larger sample sizes are needed to test for sex differences and could also benefit from including male behinds as a control condition. In addition, it might be valuable to repeat this experiment in the bonobo (*Pan Paniscus*), as this species is as closely related to us as the chimpanzee but uses sex as a way to prevent and solve conflicts, has an alpha female rather than an alpha male[44] and is known to be highly attentive towards pictures showing genitals and even pay more attention to this category than to images showing threat displays[45].

In sum, applying well-established psychological paradigms to our closest relatives represents a promising approach to providing insight into the evolution of behavior. For primates, being able to recognize each other is necessary for detecting mates. Yin’s(1969) landmark article about the ‘face inversion effect’ turned the face-literature upside-down and hundreds of articles since describe that humans process faces unlike objects. But how faces compare to another body part similar in shape, size, color and attractiveness was thus far unknown. The present study demonstrates that chimpanzees, unlike humans, show a ‘behind inversion effect’ and suggests that identity recognition ‘moved up’ from the bottom to the face in our uprightly walking species. The findings of our study suggest that over human evolution the face took over important properties shared with the primate behind and largely replaced its socio-sexual signaling function, making our species attuned to faces.

## Supporting Information

S1 Table Descriptives of all four Experiments. S1 Table shows the means and standard deviations (SD) of all four experiments for the reaction times and error rates.
(DOCX)

S2 Table Final statistical model of Experiment 1. S2 Table shows the final statistical model of Experiment 1 with human participants. Reaction times on the correct trials serve as the dependent variable.
(DOCX)

S3 Table Final statistical model of Experiment 2. S3 Table shows the final statistical model of Experiment 2 with human participants. Reaction times on the correct trials serve as the dependent variable.
(DOCX)

S4 Table Final statistical model of Experiment 3. S4 Table shows the final statistical model of Experiment 3 with chimpanzee participants. Reaction times on the correct trials serve as the dependent variable.
(DOCX)

S5 Table Final statistical model of Experiment 4. S5 Table shows the final statistical model of Experiment 4 with chimpanzee participants. Reaction times on the correct trials serve as the dependent variable.
(DOCX)
