# Supplementary material for: Getting to the Bottom of Face Processing. Species-Specific Inversion Effects for Faces and Behinds in Humans and Chimpanzees (Pan Troglodytes)
Source: PLoS One. 2016 Nov 30;11(11):e0165357. doi: 10.1371/journal.pone.0165357 (PMC5130172; doi:10.1371/journal.pone.0165357)
Supplement: S1 Table — shows the means and standard deviations (SD) of all four experiments for the reaction times and error rates. (DOCX) [file pone.0165357.s001.docx]

**Table S1. Descriptives of all four Experiments.** Table S1 shows the means and standard deviations (SD) of all four experiments for the reaction times and error rates.

|  |  |  |  |  |  |  |  |  |  |  |  |  |  |  |  |  |  |
| --- | --- | --- | --- | --- | --- | --- | --- | --- | --- | --- | --- | --- | --- | --- | --- | --- | --- |
|  |  | **Human Participants** | | | | | | | | **Chimpanzee Participants** | | | | | | | |
|  |  | **Experiment 1** | | | | **Experiment 2** | | | | **Experiment 3** | | | | **Experiment 4** | | | |
|  |  | **Reaction Time** | | **Error** | | **Reaction Time** | | **Error** | | **Reaction Time** | | **Error** | | **Reaction Time** | | **Error** | |
|  | **Orientation** | **Mean** | **SD** | **Mean** | **SD** | **Mean** | **SD** | **Mean** | **SD** | **Mean** | **SD** | **Mean** | **SD** | **Mean** | **SD** | **Mean** | **SD** |
| Human Face | Inverted | 737.41 | 263.09 | 0.09 | 0.29 | 709.35 | 221.94 | 0.08 | 0.28 | 632.05 | 172.18 | 0.22 | 0.41 | 694.42 | 182.53 | 0.20 | 0.40 |
|  | Upright | 710.15 | 281.73 | 0.05 | 0.21 | 669.48 | 194.62 | 0.07 | 0.25 | 621.02 | 160.73 | 0.19 | 0.39 | 662.58 | 179.17 | 0.18 | 0.38 |
| Human Foot | Inverted | 837.63 | 313.12 | 0.18 | 0.38 | 763.30 | 260.40 | 0.18 | 0.39 | 632.10 | 189.05 | 0.25 | 0.44 | 656.11 | 184.34 | 0.38 | 0.49 |
|  | Upright | 825.16 | 274.80 | 0.14 | 0.35 | 708.52 | 221.11 | 0.16 | 0.36 | 657.80 | 202.70 | 0.27 | 0.45 | 627.67 | 180.58 | 0.36 | 0.48 |
| Human Behind | Inverted | 807.80 | 290.21 | 0.11 | 0.31 | 869.26 | 303.27 | 0.14 | 0.35 | 579.94 | 161.85 | 0.15 | 0.36 | 683.33 | 192.99 | 0.30 | 0.46 |
|  | Upright | 804.82 | 315.09 | 0.07 | 0.25 | 860.91 | 302.36 | 0.12 | 0.32 | 558.21 | 145.55 | 0.11 | 0.32 | 691.58 | 194.21 | 0.33 | 0.47 |
| Chimpanzee Face | Inverted | 832.54 | 324.45 | 0.30 | 0.46 | 896.35 | 347.24 | 0.29 | 0.46 | 700.73 | 178.35 | 0.28 | 0.45 | 677.56 | 187.01 | 0.39 | 0.49 |
|  | Upright | 850.00 | 331.35 | 0.26 | 0.44 | 909.36 | 321.79 | 0.26 | 0.44 | 687.86 | 194.05 | 0.29 | 0.45 | 668.19 | 178.38 | 0.32 | 0.47 |
| Chimpanzee Foot | Inverted | 754.01 | 266.94 | 0.14 | 0.35 | 859.88 | 313.60 | 0.15 | 0.36 | 678.79 | 209.25 | 0.29 | 0.45 | 687.27 | 195.92 | 0.31 | 0.46 |
|  | Upright | 744.83 | 300.41 | 0.12 | 0.32 | 834.57 | 306.41 | 0.14 | 0.35 | 683.23 | 195.76 | 0.28 | 0.45 | 696.38 | 195.46 | 0.23 | 0.42 |
| Chimpanzee Behind | Inverted | 854.59 | 355.81 | 0.27 | 0.44 | 778.65 | 288.15 | 0.33 | 0.47 | 644.44 | 197.06 | 0.29 | 0.46 | 658.82 | 186.11 | 0.35 | 0.48 |
|  | Upright | 824.08 | 316.91 | 0.27 | 0.44 | 764.22 | 295.73 | 0.30 | 0.46 | 629.46 | 192.28 | 0.28 | 0.45 | 646.26 | 198.55 | 0.33 | 0.47 |
| Car | Inverted | x | x | x | x | 910.17 | 386.12 | 0.07 | 0.26 | x | x | x | x | 823.86 | 132.31 | 0.34 | 0.48 |
|  | Upright | x | x | x | x | 888.17 | 371.06 | 0.07 | 0.26 | x | x | x | x | 906.13 | 204.70 | 0.38 | 0.49 |
