# Supplementary material for: Getting to the Bottom of Face Processing. Species-Specific Inversion Effects for Faces and Behinds in Humans and Chimpanzees (Pan Troglodytes)
Source: PLoS One. 2016 Nov 30;11(11):e0165357. doi: 10.1371/journal.pone.0165357 (PMC5130172; doi:10.1371/journal.pone.0165357)
Supplement: S2 Table — shows the final statistical model of Experiment 1 with human participants. Reaction times on the correct trials serve as the dependent variable. (DOCX) [file pone.0165357.s002.docx]

**Table S2. Final statistical model of Experiment 1.** Table S2 shows the final statistical model of Experiment 1with human participants. Reaction times on the correct trials serve as the dependent variable.

Apart from the effects caused by orientation, humans were faster in recognizing human compared to chimpanzee faces (main effect ‘Stimulus Species’) and were faster in recognizing faces compared to behinds (main effect ‘Body Part’), especially when these concerned their own species (interaction ‘Body Part’ x ‘Stimulus Species’).

| **Experiment 1** |  |  |  |  |  |  |
| --- | --- | --- | --- | --- | --- | --- |
|  |  |  |  |  |  |  |
| **Fixed Effects** | **F** | **df1** | **df2** | **Sig.** |  |  |
| **Corrected Model** | 26.610 | 11 | 6.834 | 0.000 |  |  |
| **Stimulus Species** | 12.748 | 1 | 6.834 | 0.000 |  |  |
| **Body Part** | 18.931 | 2 | 6.834 | 0.000 |  |  |
| **Body Part Orientation** | 0.996 | 1 | 6.834 | 0.318 |  |  |
| **Stimulus Species * Body Part** | 110.388 | 2 | 6.834 | 0.000 |  |  |
| **Stimulus Species * Body Part Orientation** | 1.004 | 1 | 6.834 | 0.316 |  |  |
| **Body Part * Body Part Orientation** | 0.214 | 2 | 6.834 | 0.807 |  |  |
| **Stimulus Species * Body Part * Body Part Orientation** | 4.319 | 2 | 6.834 | 0.013 |  |  |
|  | **Estimate** | **SE** | **Z** | **Sig.** | **95% CI** |  |
| **Residual Effect Variance** | 0.082 | 0.001 | 58.245 | 0.000 | 0.079 | 0.084 |
| **Random Effect Var (Intercept) Participant** | 0.046 | 0.009 | 4.886 | 0.000 | 0.031 | 0.069 |
