# Supplementary material for: Getting to the Bottom of Face Processing. Species-Specific Inversion Effects for Faces and Behinds in Humans and Chimpanzees (Pan Troglodytes)
Source: PLoS One. 2016 Nov 30;11(11):e0165357. doi: 10.1371/journal.pone.0165357 (PMC5130172; doi:10.1371/journal.pone.0165357)
Supplement: S4 Table — shows the final statistical model of Experiment 3 with chimpanzee participants. Reaction times on the correct trials serve as the dependent variable. (DOCX) [file pone.0165357.s004.docx]

**Table S4. Final statistical model of Experiment 3.** Table S4 shows the final statistical model of Experiment 3 in chimpanzee participants. Reaction times on the correct trials serve as the dependent variable.

Apart from the effects caused by orientation, chimpanzees were faster in recognizing behinds compared to faces (main effect ‘Body Part’) and faster in recognizing human compared to chimpanzee faces (main effect ‘Stimulus Species’).

|  |  |  |  |  |  |  |
| --- | --- | --- | --- | --- | --- | --- |
|  |  |  |  |  |  |  |
| **Experiment 3** |  |  |  |  |  |  |
|  |  |  |  |  |  |  |
| **Fixed Effects** | **F** | **df1** | **df2** | **Sig.** |  |  |
| **Corrected Model** | 21.424 | 8 | 2.600 | 0.000 |  |  |
| **Stimulus Species** | 80.703 | 1 | 2.600 | 0.000 |  |  |
| **Body Part** | 33.321 | 2 | 2.600 | 0.000 |  |  |
| **Body Part Orientation** | 1.206 | 1 | 2.600 | 0.272 |  |  |
| **Stimulus Species * Body Part** | 2.375 | 2 | 2.600 | 0.093 |  |  |
| **Stimulus Species * Body Part Orientation** | x | x | x | x |  |  |
| **Body Part * Body Part Orientation** | 2.809 | 2 | 2.600 | 0.060 |  |  |
| **Stimulus Species * Body Part * Body Part Orientation** | x | x | x | x |  |  |
|  | **Estimate** | **SE** | **Z** | **Sig.** | **95% CI** |  |
| **Residual Effect Variance** | 0.065 | 0.002 | 35.710 | 0.000 | 0.061 | 0.068 |
| **Random Effect Var (Intercept) Participant** | 0.015 | 0.011 | 1.322 | 0.186 | 0.003 | 0.066 |
| **Random Effect Var (Intercept) Participant * Session** | 0.004 | 0.002 | 2.432 | 0.015 | 0.002 | 0.01 |
